# Supplementary material for: MetaRibo-Seq measures translation in microbiomes
Source: Nat Commun. 2020 Jun 29;11:3268. doi: 10.1038/s41467-020-17081-z (PMC7324362; doi:10.1038/s41467-020-17081-z)
Supplement: Supplementary file 10 — Supplementary Data 7 [file 41467_2020_17081_MOESM10_ESM.zip › File2/Confidence_High_Taxonomy/220632_out.krona.html]

Javascript must be enabled to view this page.

members
magnitude
magnitudeUnassigned
count
unassigned
taxon
rank

220632\_out

361

2
361
superkingdom

1239
3
phylum

class
2
186801

order
2
186802

family
1
186803

1

SRS147377\_contig\_number\_542
39491
species

541000
1
family

1
216851
genus

853

SRS014412\_contig\_number\_3257
1
species

1263019
1

SRS148721\_contig\_number\_39442
species

358
976
phylum

class
200643
358

358
171549
order

171550
1
family

239759
1
genus

species

SRS144603\_contig\_number\_2548
1
1262694

815
355
family


SRS013158\_contig\_number\_4541SRS013521\_contig\_number\_137SRS013951\_contig\_number\_39775SRS014235\_contig\_number\_contig-100\_7040.193051SRS014683\_contig\_number\_18491SRS015264\_contig\_number\_437SRS015960\_contig\_number\_22472SRS016989\_contig\_number\_14408SRS017103\_contig\_number\_43089SRS017191\_contig\_number\_14324SRS017701\_contig\_number\_20159SRS017821\_contig\_number\_9666SRS018427\_contig\_number\_12620SRS019161\_contig\_number\_13553SRS019601\_contig\_number\_contig-100\_895.157868SRS019910\_contig\_number\_20995SRS023583\_contig\_number\_17307SRS043701\_contig\_number\_3799SRS045004\_contig\_number\_4709SRS045645\_contig\_number\_2356SRS045826\_contig\_number\_contig-100\_4277.117761SRS048870\_contig\_number\_13574SRS049446\_contig\_number\_1212SRS049712\_contig\_number\_21796SRS049896\_contig\_number\_26243SRS049900\_contig\_number\_4141SRS049995\_contig\_number\_11158SRS050299\_contig\_number\_9745SRS052027\_contig\_number\_14152SRS053649\_contig\_number\_12460SRS056259\_contig\_number\_22777SRS063040\_contig\_number\_contig-100\_566.17659SRS074586\_contig\_number\_4855SRS075821\_contig\_number\_13223SRS075878\_contig\_number\_18815SRS077127\_contig\_number\_5694SRS077392\_contig\_number\_contig-100\_3039.238888SRS077849\_contig\_number\_3404SRS097889\_contig\_number\_3814SRS098073\_contig\_number\_2990SRS098571\_contig\_number\_19390SRS098644\_contig\_number\_13349SRS100907\_contig\_number\_contig-100\_893.63335SRS103987\_contig\_number\_6042SRS1041091\_contig\_number\_22767SRS1041129\_contig\_number\_9974SRS1041133\_contig\_number\_25483SRS1054928\_contig\_number\_7190SRS1055095\_contig\_number\_545SRS140492\_contig\_number\_14178SRS143466\_contig\_number\_20194SRS143780\_contig\_number\_5262SRS143780\_contig\_number\_contig-100\_1249.35231SRS143876\_contig\_number\_13272SRS143895\_contig\_number\_42153SRS144135\_contig\_number\_33932SRS146812\_contig\_number\_50724SRS147022\_contig\_number\_4790SRS147346\_contig\_number\_39352SRS147557\_contig\_number\_contig-100\_2700.198250SRS148511\_contig\_number\_contig-100\_3787.105005SRS149325\_contig\_number\_3540SRS893230\_contig\_number\_contig-100\_3082.67728SRS893342\_contig\_number\_contig-100\_3610.59786
355
816
genus
64

species
2

SRS013382\_contig\_number\_442SRS018836\_contig\_number\_11984
2292944


SRS075398\_contig\_number\_383
1
28116
species

species
46506
2

SRS1041157\_contig\_number\_6956SRS143372\_contig\_number\_64

671267
2

SRS1041039\_contig\_number\_26129SRS1041136\_contig\_number\_23617
species

species
1

SRS014855\_contig\_number\_14073
1263042

species
47678

SRS023526\_contig\_number\_9692SRS052697\_contig\_number\_236SRS142599\_contig\_number\_14853SRS144362\_contig\_number\_9027
4


SRS023971\_contig\_number\_4236
1
1897052
species

species
1

SRS044535\_contig\_number\_10599
310297

1

SRS146764\_contig\_number\_11365
1262750
species

species
1262735
1

SRS053398\_contig\_number\_3895

species

SRS011084\_contig\_number\_20933SRS011134\_contig\_number\_16436SRS011239\_contig\_number\_18332SRS011302\_contig\_number\_20686SRS012849\_contig\_number\_contig-100\_3327.178990SRS012902\_contig\_number\_167SRS012969\_contig\_number\_10091SRS013638\_contig\_number\_18792SRS013687\_contig\_number\_31730SRS013965\_contig\_number\_35128SRS014287\_contig\_number\_10291SRS014313\_contig\_number\_3603SRS014459\_contig\_number\_16891SRS014534\_contig\_number\_553SRS014736\_contig\_number\_7088SRS014948\_contig\_number\_contig-100\_1526.127862SRS014979\_contig\_number\_29751SRS015065\_contig\_number\_26474SRS015190\_contig\_number\_14170SRS015217\_contig\_number\_15462SRS015369\_contig\_number\_5566SRS015431\_contig\_number\_44723SRS015578\_contig\_number\_27389SRS015579\_contig\_number\_24493SRS015663\_contig\_number\_24628SRS015694\_contig\_number\_16033SRS015782\_contig\_number\_27135SRS015794\_contig\_number\_contig-100\_6403.64105SRS015854\_contig\_number\_3665SRS016056\_contig\_number\_240SRS016095\_contig\_number\_1048SRS016203\_contig\_number\_1964SRS016335\_contig\_number\_44777SRS016495\_contig\_number\_contig-100\_1186.114396SRS016629\_contig\_number\_contig-100\_184.34586SRS016840\_contig\_number\_1249SRS016897\_contig\_number\_141SRS016954\_contig\_number\_12985SRS017247\_contig\_number\_5932SRS017375\_contig\_number\_contig-100\_77.18082SRS017433\_contig\_number\_8928SRS017521\_contig\_number\_15769SRS017745\_contig\_number\_203SRS018351\_contig\_number\_9218SRS018623\_contig\_number\_4739SRS018817\_contig\_number\_9511SRS018888\_contig\_number\_5105SRS018936\_contig\_number\_8458SRS019030\_contig\_number\_631SRS019068\_contig\_number\_26057SRS019178\_contig\_number\_116SRS019267\_contig\_number\_448SRS019381\_contig\_number\_6125SRS019397\_contig\_number\_4025SRS019445\_contig\_number\_14656SRS019496\_contig\_number\_1212SRS019693\_contig\_number\_7101SRS019808\_contig\_number\_20826SRS019968\_contig\_number\_8729SRS020328\_contig\_number\_3981SRS020394\_contig\_number\_13081SRS020508\_contig\_number\_143SRS020622\_contig\_number\_56SRS021153\_contig\_number\_1419SRS021219\_contig\_number\_6112SRS022137\_contig\_number\_contig-100\_1357.119590SRS022524\_contig\_number\_15312SRS022609\_contig\_number\_44548SRS023914\_contig\_number\_13697SRS024009\_contig\_number\_12060SRS024331\_contig\_number\_22895SRS024625\_contig\_number\_740SRS042284\_contig\_number\_2087SRS042966\_contig\_number\_10658SRS043411\_contig\_number\_2845SRS043768\_contig\_number\_30015SRS045244\_contig\_number\_149SRS046068\_contig\_number\_4470SRS046369\_contig\_number\_2657SRS046502\_contig\_number\_12660SRS046810\_contig\_number\_5SRS047014\_contig\_number\_37713SRS047044\_contig\_number\_17704SRS047433\_contig\_number\_1883SRS048164\_contig\_number\_30702SRS049402\_contig\_number\_2303SRS049773\_contig\_number\_16668SRS049959\_contig\_number\_26282SRS050026\_contig\_number\_204SRS050422\_contig\_number\_18350SRS050520\_contig\_number\_248SRS050801\_contig\_number\_2704SRS050925\_contig\_number\_25628SRS050974\_contig\_number\_968SRS050998\_contig\_number\_11427SRS051031\_contig\_number\_761SRS051610\_contig\_number\_8325SRS051882\_contig\_number\_3553SRS052576\_contig\_number\_160SRS053087\_contig\_number\_2564SRS053214\_contig\_number\_12017SRS053356\_contig\_number\_14466SRS054059\_contig\_number\_contig-100\_7.45377SRS054905\_contig\_number\_14124SRS055017\_contig\_number\_27130SRS055982\_contig\_number\_15224SRS056273\_contig\_number\_28795SRS056537\_contig\_number\_32200SRS057480\_contig\_number\_8258SRS058145\_contig\_number\_2047SRS058268\_contig\_number\_contig-100\_23.24SRS058723\_contig\_number\_1225SRS062427\_contig\_number\_1003SRS062701\_contig\_number\_1191SRS063190\_contig\_number\_5762SRS063370\_contig\_number\_716SRS063489\_contig\_number\_1990SRS063518\_contig\_number\_11055SRS063813\_contig\_number\_1428SRS063928\_contig\_number\_716SRS064232\_contig\_number\_contig-100\_1794.39986SRS064645\_contig\_number\_740SRS064973\_contig\_number\_576SRS065176\_contig\_number\_2090SRS065397\_contig\_number\_12951SRS065504\_contig\_number\_21737SRS074670\_contig\_number\_178SRS074964\_contig\_number\_2238SRS075078\_contig\_number\_10392SRS075716\_contig\_number\_2064SRS075773\_contig\_number\_36219SRS076756\_contig\_number\_9632SRS076876\_contig\_number\_488SRS076929\_contig\_number\_12328SRS076976\_contig\_number\_3534SRS077194\_contig\_number\_12640SRS077231\_contig\_number\_12351SRS077294\_contig\_number\_3680SRS077335\_contig\_number\_5338SRS077454\_contig\_number\_3010SRS077502\_contig\_number\_14319SRS077589\_contig\_number\_1116SRS077641\_contig\_number\_22873SRS077730\_contig\_number\_1941SRS077991\_contig\_number\_contig-100\_21.23928SRS078242\_contig\_number\_7006SRS078665\_contig\_number\_789SRS097920\_contig\_number\_20600SRS097958\_contig\_number\_15151SRS098061\_contig\_number\_8448SRS098827\_contig\_number\_1485SRS098881\_contig\_number\_1500SRS100021\_contig\_number\_5821SRS101433\_contig\_number\_11398SRS101441\_contig\_number\_contig-100\_661.661SRS104036\_contig\_number\_7610SRS104084\_contig\_number\_8893SRS1041031\_contig\_number\_25075SRS1041037\_contig\_number\_11227SRS1041090\_contig\_number\_5457SRS1041092\_contig\_number\_2337SRS1041116\_contig\_number\_3833SRS1041130\_contig\_number\_828SRS1041134\_contig\_number\_11833SRS1041137\_contig\_number\_7327SRS1041139\_contig\_number\_938SRS1041140\_contig\_number\_14153SRS1041141\_contig\_number\_44SRS1041142\_contig\_number\_53SRS1041143\_contig\_number\_180SRS1041144\_contig\_number\_86SRS1041147\_contig\_number\_1794SRS1041164\_contig\_number\_1460SRS104327\_contig\_number\_contig-100\_985.43518SRS104400\_contig\_number\_3965SRS104485\_contig\_number\_86SRS104693\_contig\_number\_8387SRS104975\_contig\_number\_1239SRS105082\_contig\_number\_3765SRS105153\_contig\_number\_contig-100\_7492.185048SRS1054716\_contig\_number\_4532SRS1055022\_contig\_number\_11623SRS1055038\_contig\_number\_610SRS1055049\_contig\_number\_11469SRS1055056\_contig\_number\_6860SRS1055069\_contig\_number\_243SRS1055076\_contig\_number\_contig-100\_1851.91140SRS1055083\_contig\_number\_452SRS140513\_contig\_number\_12130SRS142503\_contig\_number\_34171SRS142542\_contig\_number\_4295SRS142781\_contig\_number\_410SRS142890\_contig\_number\_38836SRS142923\_contig\_number\_20166SRS142980\_contig\_number\_4270SRS143148\_contig\_number\_12505SRS143181\_contig\_number\_13573SRS143417\_contig\_number\_17012SRS143722\_contig\_number\_2466SRS143991\_contig\_number\_154SRS144183\_contig\_number\_41297SRS144297\_contig\_number\_14443SRS144506\_contig\_number\_35433SRS144537\_contig\_number\_49543SRS144714\_contig\_number\_28541SRS144753\_contig\_number\_12254SRS145308\_contig\_number\_30SRS145497\_contig\_number\_11609SRS146813\_contig\_number\_17985SRS147039\_contig\_number\_377SRS147425\_contig\_number\_5054SRS147614\_contig\_number\_2358SRS147653\_contig\_number\_8204SRS147766\_contig\_number\_13523SRS147977\_contig\_number\_3010SRS148159\_contig\_number\_55745SRS148196\_contig\_number\_40545SRS148253\_contig\_number\_12667SRS148424\_contig\_number\_30049SRS148784\_contig\_number\_13851SRS148970\_contig\_number\_198SRS149075\_contig\_number\_2940SRS149181\_contig\_number\_30057SRS149244\_contig\_number\_1453SRS150029\_contig\_number\_contig-100\_5415.97062SRS892882\_contig\_number\_979SRS893170\_contig\_number\_7968SRS893172\_contig\_number\_52SRS893187\_contig\_number\_12824SRS893252\_contig\_number\_1240SRS893253\_contig\_number\_225SRS893256\_contig\_number\_677SRS893259\_contig\_number\_6393SRS893270\_contig\_number\_22989SRS893279\_contig\_number\_1418SRS893285\_contig\_number\_841SRS893288\_contig\_number\_752SRS893292\_contig\_number\_8963SRS893295\_contig\_number\_contig-100\_2217.80222SRS893300\_contig\_number\_13077SRS893327\_contig\_number\_4128SRS893341\_contig\_number\_5414SRS893358\_contig\_number\_2378SRS893366\_contig\_number\_7735SRS893373\_contig\_number\_21708SRS893378\_contig\_number\_10274SRS893380\_contig\_number\_618SRS893382\_contig\_number\_263SRS893383\_contig\_number\_20717SRS971275\_contig\_number\_35871
250
821

2

SRS050752\_contig\_number\_19278SRS893369\_contig\_number\_58
1638779
species

species
204516
2

SRS021948\_contig\_number\_13303SRS078176\_contig\_number\_11467

species
3

SRS021484\_contig\_number\_18795SRS050941\_contig\_number\_11088SRS101376\_contig\_number\_4542
29523

species
291644
2

SRS016753\_contig\_number\_2762SRS045739\_contig\_number\_5367

310300

SRS077552\_contig\_number\_12137SRS1041036\_contig\_number\_1449
2
species

species

SRS016132\_contig\_number\_330SRS017859\_contig\_number\_1578SRS024075\_contig\_number\_187SRS075984\_contig\_number\_599
4
246787

1262754

SRS1041138\_contig\_number\_9072
1
species

species

SRS023829\_contig\_number\_48SRS143342\_contig\_number\_9292
2
817

820

SRS053335\_contig\_number\_13128SRS077086\_contig\_number\_3593SRS143085\_contig\_number\_922SRS148817\_contig\_number\_1814
4
species

species
1263040
1

SRS054352\_contig\_number\_4830

species
818

SRS024492\_contig\_number\_126
1

species

SRS1041118\_contig\_number\_440
1
329854

family
171552
1

577309
1
genus


SRS017307\_contig\_number\_12515
1
454154
species

1
1853231
family

283168
1
genus

species
1637502
1

SRS062654\_contig\_number\_99
